# The Automatic Conservative: Ideology-Based Attentional Asymmetries in the Processing of Valenced Information

**DOI:** 10.1371/journal.pone.0026456

**Published:** 2011-11-09

**Authors:** Luciana Carraro, Luigi Castelli, Claudia Macchiella

**Affiliations:** Dipartimento di Psicologia dello Sviluppo e della Socializzazione, University of Padova, Padova, Italy; University of Bologna, Italy

## Abstract

Research has widely explored the differences between conservatives and liberals, and it has been also recently demonstrated that conservatives display different reactions toward valenced stimuli. However, previous studies have not yet fully illuminated the cognitive underpinnings of these differences. In the current work, we argued that political ideology is related to selective attention processes, so that negative stimuli are more likely to automatically grab the attention of conservatives as compared to liberals. In Experiment 1, we demonstrated that negative (vs. positive) information impaired the performance of conservatives, more than liberals, in an Emotional Stroop Task. This finding was confirmed in Experiment 2 and in Experiment 3 employing a Dot-Probe Task, demonstrating that threatening stimuli were more likely to attract the attention of conservatives. Overall, results support the conclusion that people embracing conservative views of the world display an automatic selective attention for negative stimuli.

## Introduction

*“All people are born alike - except Republicans and Democrats”* (Groucho Marx)

As remarked by Groucho Marx, political conservatives and liberals often present dramatic differences. Research in the last decades has widely investigated the distinguishing features of these opposing ideologies in various domains. For instance, conservatives and liberals tend to display different personality profiles (e.g. [1], [2]), and even facial features [3]. In addition, political ideology is related to differences in cognitive styles (e.g., need for closure, structure, and order) [4]. For instance, conservatives show greater neurocognitive sensitivity to changes in habitual patterns of response [5], tend to more rigidly organize their living and working spaces [6], and express preferences for simple and easily interpretable pieces of art [7], whereas liberals display more integrative complexity and tolerance of ambiguity [4].

Remarkable differences between conservatives and liberals also emerge in relation to the perception of the world as dangerous and threatening [8], [9]. Interestingly, structural MRI data demonstrated that conservatives have an increased gray matter volume of the right amygdala [10], a brain structure involved in the processing of threatening information [11]. This suggests that individuals embracing conservative political views might be more sensitive to signals of threat, and display avoidance regulatory strategies, that is an orientation focused on the prevention of negative outcomes at both a personal and group level [12], [13]. In line with this idea, it has been recently found that conservatives display higher changes in skin conductance, as compared to liberals, when they are presented with threatening stimuli (e.g., a bloody face) [14]. A further relevant demonstration has been provided by Shook and Fazio [15] who nicely showed that, conservatives and liberals explore novel situations differently, with the former being more cautious and more likely to display learning asymmetries, namely a tendency to learn negative items relatively better than positive items. A third line of research [16] indicates that conservatives are more likely to interpret ambiguous facial stimuli as expressing threatening emotions. In impression formation tasks, conservatives, as compared to liberals, give also more weight to negative as compared to positive information [17]. Overall, these findings consistently make evident the existence of a link between political ideology and the processing of valenced information. However, previous studies have not yet fully enlightened the cognitive underpinnings of the differential reactions to positive and negative stimuli as a function of political ideology. Indeed, the aforementioned results might stem from intentional and conscious processes that prioritize either positive or negative information. It should be noted that in the work by Oxley and colleagues [14], changes in skin conductance were assessed after prolonged exposure to either threatening or non-threatening stimuli, whereas startle-blink responses were related to sudden noises and not to intrinsically threatening stimuli.

Therefore, it is critical to get underneath the observed effects described in literature and disambiguate whether they are based on higher-level cognitive processes or basic attentional processes. In the current studies, we explored whether conservatives and liberals automatically respond in a different way to positive and negative stimuli. More specifically, we tested the hypothesis that conservatives, as compared to liberals, are characterized by stronger automatic selective attention toward negative stimuli.

In Experiment 1, we relied on a modified version of the Stroop task [18]. This experimental paradigm allows us to identify the extent to which an irrelevant stimulus feature attracts the attention and interferes with the execution of the primary task. Participants were required to define the color of the font of positive and negative words, so that to assess how the irrelevant stimulus valence automatically attracted participants' attention (i.e., Emotional Stroop Task) [19]–[20]. As mentioned above, we predicted that conservative political views would correlate with the attention-grabbing power of negative as compared to positive information.

## Methods

### Experiment 1

#### Participants

Forty-five students (43 female; age *M* = 20.16, *SD* = 3.96) participated in the experimental study in exchange of course credits. The experiment was conducted in accordance with the guidelines laid down in the Declaration of Helsinki and with the indications of the local ethical committee. Participants were fully informed about the structure of the study and their rights, and provided an oral consent prior to taking part in the experiment.

#### Procedure

Political ideology was assessed during a class session some weeks before participation to the main study. Participants (N = 166) were asked to express their agreement (from 1 = “not at all” to 7 = “very much”) towards 6 social issues (i.e., reduction of immigration, abortion, medically assisted procreation, homosexual marriage, use of arms for personal defense, adoption by homosexual couples; α = .70). Responses were rescaled so that higher scores corresponded to more conservative views (*Mean of the experimental sample* = 3.67, *SD* = 1.11). Data from a different sample (N = 40) showed that responses to the ideology scale are correlated with political affiliation as measured on a 10 cm continuum (from left-wing to right-wing), *r*(40) = .62, *p*<.001.

In the laboratory study, participants performed an Emotional Stroop Task. They were presented with 20 positive (e.g., love, peace, nice, honesty, friendship, harmony, joy, pleasure, paradise, quiet, sincerity, happiness, wonderful, balanced, peacefulness, order, stability, freedom, security, serenity) and 20 negative words (e.g., anger, hate, vomit, horrible, disorder, disgust, horror, contempt, pain, accident, disaster, suffering, sickness, dirty, repulsion, terror, awful, evil, threatening, grief); half of both positive and negative words were printed in blue, whereas the other half was printed in red. What specific words were printed in red or blue was counterbalanced across participants. Overall, participants were presented with 200 trials in a random order and they were asked to quickly and accurately categorize the color in which the words were written while ignoring their meaning. If the valence of the word automatically attracts the attention, the performance in the color-naming task is expected to be impaired. Each word was visible at the centre of the screen until response (ISI = 150 ms). At the top of the screen, two black labels (e.g., “D = red” and “K = blue” or vice-versa) always reminded the meaning of the response keys. Next, participants were asked to evaluate the valence of the 40 words (from 1 = “extremely positive” to 7 = “extremely negative”). Finally, they were thanked and fully debriefed during a class lesson.

### Experiment 2

#### Participants

Forty students (16 female) aged between 20 and 28 years (*M* = 22.40, *SD* = 1.81) participated in the study on a voluntary basis. The experiment was conducted in accordance with the guidelines laid down in the Declaration of Helsinki and with the indications of the local ethical committee. Participants were fully informed about the structure of the study and their rights, and provided a written consent prior to taking part in the experiment. It was stressed that they could freely leave the study at any time, but all participants completed it.

#### Procedure

Participants initially reported their agreement (from 1 = “not at all” to 7 = “very much”) towards 10 social issues (i.e., reduction of immigration, abortion, medically assisted procreation, homosexual marriage, adoption by homosexual couples, legalization of soft drugs, euthanasia, globalization, use of stem cells, environmental pollution; α = .78). Responses were rescaled so that higher scores corresponded to more conservative views (*M* = 3.34, *SD* = .94).

Afterwards, the Dot-Probe Task was introduced. Participants were instructed that on each trial, two different pictures would briefly appear (i.e., 500 ms) on the computer screen one next to the other (one on the left- and one on the right-side of the computer screen, followed by a small grey dot on either the left- or right-side of the screen. On each trial a positive and a negative image were simultaneously presented. Overall, 8 positive (number 1440, 1710, 2070, 4626, 5030, 5779, 5831, 7325; e.g., a flower, a baby) and 8 negative images (number 1300, 1930, 2120, 2811, 5970, 6560, 8480, 9440; e.g., an hurricane; a shark) from the International Affective Picture System [21] were used. Negative pictures were slightly more arousing than positive pictures, but the difference was not statistically significant. Negative and positive pictures randomly appeared on the right- or left-side of the screen. Overall, participants went through 64 trials, and the dot appeared in the same spatial location of the negative image in half of the trials. Participants were asked to quickly determine the spatial location of the dot by pressing a key on the computer keyboard (“D” and “K” when the dot was on the left- or right-side, respectively).

Finally, participants rated the valence of each picture (from 1 = “extremely positive” to 7 = “extremely negative”), before being thanked and debriefed.

### Experiment 3

#### Participants

Twenty-two students (17 female) aged between 18 and 23 years (*M* = 19.41, *SD* = 1.01) participated in the study in exchange of course credits. The experiment was conducted in accordance with the guidelines laid down in the Declaration of Helsinki and with the indications of the local ethical committee. Participants were fully informed about the structure of the study and their rights, and provided a written consent prior to taking part in the experiment.

#### Procedure

A couple of weeks before participation to the laboratory study, participants were asked to complete a questionnaire during a class session. First of all, participants (N = 165) were asked to express their agreement (from 1 = “not at all” to 7 = “very much”) towards 15 social and economic issues (i.e., reduction of immigration, abortion, medically assisted procreation, homosexual marriage, adoption by homosexual couples, globalization, soft drugs, industrial relations, tax for high income, stem cells, pollution, equality in the distribution of resources, euthanasia, privatization, federalism; α = .77). Responses were rescaled so that higher scores corresponded to more conservative political ideology (*Mean of the experimental sample* = 3.16, *SD* = .72). Two additional measures were included in the questionnaire. One 15-item measure assessed Need for Closure [22] and responses had to be provided along 7-point Likert scales (α = .72, *M* = 3.82, *SD* = .85; higher scores indicated high need for closure). The second measure comprised 18 items and assessed Need for Cognition [23], [24]; responses were provided along 4-point scales (α = .77, *M* = 2.92, *SD* = .36; high scores indicated high need for cognition). As expected, overall political ideology was correlated with both need for closure, *r*(165) = .19, *p* = .01, and need for cognition, *r*(165) = −.18, *p* = 02. However, need for closure and need for cognition were not correlated, *r*(165) = −.04, *ns.*

In the laboratory study, participants performed the very same Dot-Probe Task described in Experiment 2 with positive and negative images simultaneously presented on the computer screen. Finally, participants were thanked and fully debriefed during a class session.

## Results

### Experiment 1

#### Emotional Stroop Task

**Figure 1:**
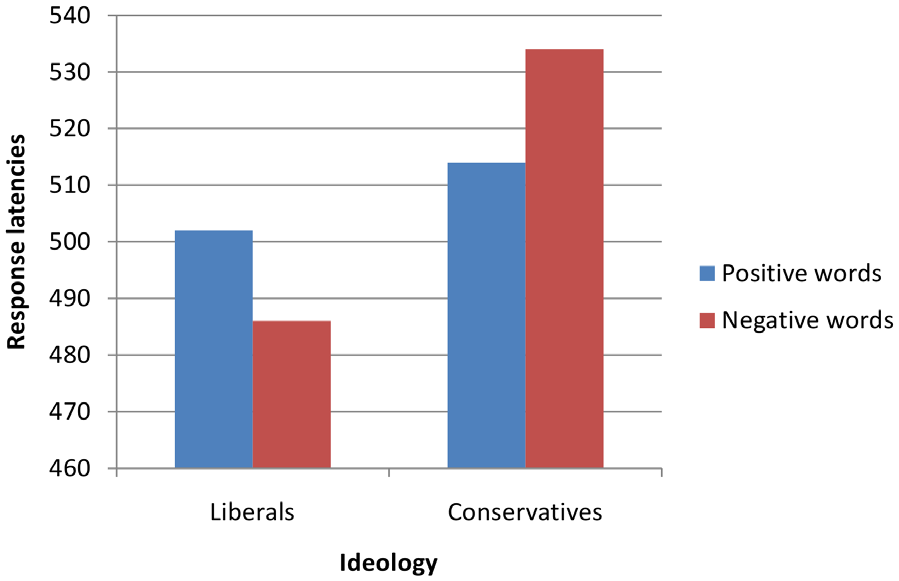
Response latencies to positive and negative words as a function of participants' ideology (Experiment 1).

For each participant we calculated the difference between the mean latency of correct responses for negative (M = 508 ms) and positive words (M = 511 ms; overall less than 3% of errors), so that positive values indicated slower responses to negative as compared to positive words. In order to test our main hypothesis, we correlated political ideology and this difference score, showing a significant association, *r*(45) = .38, *p* = .009. This indicates that more conservative individuals had a stronger automatic vigilance toward negative as compared to positive stimuli. Next, we assessed the specific association between political ideology and responses to the two types of words. Responses to negative and positive words were regressed to ideology, and whereas the former were positively associated, β = .81, *t*(44) = 3.01, *p* = .004, the latter were negatively associated, β = −.68, *t*(44) = −2.54, *p* = .015. These results suggest that conservatives, as compared to liberals, are more distracted by negative stimuli but at the same time they show faster responses to positive stimuli. In order to further explore these findings, participants were divided in two groups (i.e., split-half) on the basis of their responses to the ideology scale. Next a 2 (valence of the words)×2 (ideology: liberal vs conservative) analysis of variance was performed, with the first factor within participants and the second factor between-participants. Results showed a significant interaction effect, *F*(1, 43) = 5.23, *p*<.05 (see Figure 1). In particular, liberals were slightly faster to respond to negative than positive words, but the effect was not statistically significant, *p* = .25. In contrast, conservatives were significantly slower in their responses to negative as compared to positive words, *t*(22) = 2.36, *p*<.05. Post-hoc tests also showed that the responses of liberals and conservatives to positive items were not different, *p* = . 69, whereas conservatives tended to be slower than liberals when responding to negative items, *p*<.05, one-tailed.

#### Explicit evaluations

First, we calculated the mean for both negative (α = .89, *M* = 5.6, *SD* = .62) and positive words (α = .81, *M* = 1.68, *SD* = .40). Results showed that political ideology was not a significant predictor of the evaluation of negative words, β = .20, *t*(44) = 1.35, *p* = .18, of positive words, β = .10, *t*(44) = .64, *p* = .52, or of the difference between the evaluation of the two types of words, β = .11, *t*(44) = .49, *p* = .49. The ideology-based difference emerged from the Emotional Stroop Task cannot thus be explained by differences in the appraisal of the stimuli.

Results from Experiment 1 clearly showed that conservatives, as compared to liberals, were more likely to automatically direct their attentional resources away from the attended task and allocate them to negative stimuli. Participants' ideological standing was not related to the explicit evaluation of the stimuli, but it had a direct reflection on the automatic reaction to those stimuli. It is interesting to note that political ideology was assessed by a different experimenter in a different context, several weeks before the experimental session, and therefore it was not situationally activated. This suggests that conservatives and liberals display chronic differences in their automatic allocation of attentional resources.

In Experiment 2, we further tested this hypothesis by exploring the regulation of visual attention. In the former study, participants were presented with only a single item and the interference of a task-irrelevant stimulus dimension (i.e., valence) was assessed, whereas we here briefly presented participants with two stimuli simultaneously. We employed a Dot-Probe procedure [19], [25] in which participants had to detect the spatial location of a target which suddenly appeared where either a positive or negative stimulus had been initially shown. Whereas in Stroop-like tasks slower responses indicate that the stimulus has grabbed the attention of the participant thus interfering with color naming, the logic of the Dot-Probe task is very different. Indeed, fast response latencies in this task indicate that participants' attention was already oriented toward the stimulus that actually masked the probe. In contrast, long response latencies signal that participants' attention had been oriented to the stimulus that had not obscured the probe. We predicted that conservatives, as compared to liberals, would be more likely attracted by negative stimuli, and would thus be faster to detect a probe appearing in the same spatial location of such negative stimuli.

### Experiment 2

#### Dot-Probe Task

The mean latencies of correct responses (overall 3.1% of errors) when the dot followed negative and positive pictures were separately calculated (*M*s = 452 ms in both cases). A difference score was computed so that positive values indicated slower responses after positive as compared to negative pictures. As expected, this index was positively correlated with participants' ideology, *r*(40) = .36, *p* = .023. The more a participant embraced conservative views of the world, the more he/she was also faster in responding to the dot appearing in the same spatial location of negative images than positive images. This is consistent with the idea that conservatives display an attentional bias for negative stimuli. Next, response latencies to dots following negative and positive images were regressed to ideology: the former were negatively associated, β = −.80, *t*(39) = −2.04, *p* = .049, whereas the latter were positively associated, β = .95, *t*(39) = 2.42, *p* = .02. As in Experiment 1, participants were divided in two groups on the basis of their responses to the ideology scale and response latencies were submitted to a 2 (valence of the images)×2 (ideology: liberal vs conservative) analysis of variance. The predicted interaction effect did not reach the conventional level of significance, *F*(1, 38) = 2.22, *p* = .14. However, the responses of liberals were slightly faster when the dot was obscured by a positive (*M* = 444, *SD* = 81) rather than a negative image (*M* = 451, *SD* = 84). The pattern of responses was reversed in the case of conservatives (positive images: *M* = 460, *SD* = 68; negative images: *M* = 453, *SD* = 58).

#### Explicit Evaluation

The evaluation of positive (*M* = 6.05, *SD* = .66) and negative images (*M* = 2.29, *SD* = .82) was calculated. Results showed that political ideology was not related to the evaluation of negative, β = −.22, *t*(39) = −1.38, *p* = .17, or positive images, β = −.08, *t*(39) = −.52, *p* = .60, nor was it related to the difference between the evaluation of the two types of images, β = .12, *t*(39) = .67, *p* = .51. As in Experiment 1, the ideology-based difference emerged from the Dot-Probe Task cannot be explained by differences in the appraisal of the stimuli.

Results from Experiment 2 clearly showed that conservatives, as compared to liberals, when simultaneously presented with a negative and a positive stimulus, were more likely to automatically direct their attention toward the negative one. Conservatives and liberals provided similar explicit evaluations about the valence of the presented images, but their automatic attention was differently affected by the valence of stimuli. In Experiment 3 we further attempted to replicate this pattern of findings while controlling for other potentially relevant variables. Indeed, political ideology is currently conceived as a set of beliefs that enables us to fulfill relational, epistemic, and existential needs [26]. It is thus a higher-order construct that subsumes several more specific motivational drives. For instance, conservatives usually report higher levels of need for closure [27] but lower levels of need for cognition [23], [4]. Thus, we assessed liberals and conservatives automatic attentional processes while taking into account eventual differences related to those other motivational factors. More specifically, in Experiment 3 participants were asked to complete the very same task presented in Experiment 2 and their need for closure and need for cognition were also assessed. Moreover, in Experiment 3 the measure of political ideology comprised items related to both social and economic issues [28], [29]. We predicted that, as demonstrated in Experiment 2, conservatives, as compared to liberals, would be more likely attracted by negative stimuli, and would thus be faster to detect a probe appearing in the same spatial location of such negative stimuli, even when other specific individual differences (i.e., need for closure and need for cognition) are taken under control.

### Experiment 3

#### Dot-Probe Task

**Figure 2:**
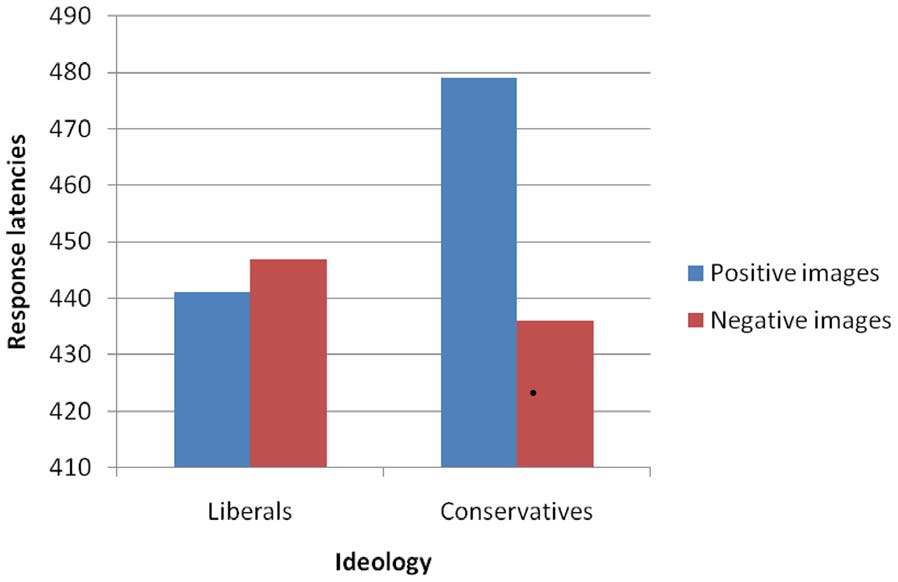
Responses in the Dot-Probe Task as a function of the valence of the image that obscured the dot and participants' ideology (Experiment 3).

As in Experiment 2, for each participant the mean latencies of correct responses when the dot followed negative (*M* = 441 ms, *SD* = 55) and positive (*M* = 462 ms, *SD* = 82) images were separately calculated. In a linear regression analysis, political ideology was negatively related to latencies when the dot followed a negative image, β = −.67, *t*(21) = −2.44, *p* = .025, and positively related to the latencies when the dot followed a positive image, β = .76, *t*(21) = 2.77, *p* = .012. This pattern was confirmed by a 2 (valence of the images)×2 (ideology: liberal vs conservative) analysis of variance in which ideology was considered as a categorical variable. Indeed, a significant interaction emerged, *F*(1, 20) = 4.89, *p*<.05 (see Figure 2). Whereas the responses of liberals did not differ as a function of the valence of the image that obscured the dot (*p*>.48), conservatives were significantly faster when the dot appeared in the same spatial location of a negative rather than positive image, *p*<.05.

Next, a difference score was computed in such a way that positive scores indicated faster responses after negative as compared to positive pictures. A preliminary analysis showed that this score was significantly correlated with participants' ideology, *r*(22) = .53, *p* = .013, thus replicating what observed in Experiment 2. In a linear regression analysis, this index was then entered as a dependent variable whereas political ideology, need for cognition and need for closure were simultaneously entered as independent variables. As expected, only political ideology emerged to be a significant predictor of the performance in the Dot-Probe Task, β = .51, *t*(21) = 2.61, *p* = .018. More specifically conservatives were faster when the dot followed a negative images as compared to positive images. The effects of need for closure, β = .16, *t*(21) = .80, and need for cognition, β = −.11, *t*(21) = −.56, were not significant. Separate scores about the economic and social issues addressed in the ideology scale were also calculated and they proved to be weakly correlated, *r* = .33, *p* = .12. Both the social, *r* = .48, *p*<.05 and the economic dimensions scores, *r* = .37, *p* = .09, were associated to the difference scores between responses to positive and negative stimuli.

## Discussion

Research has widely explored the differences between conservatives and liberals, and dozens of studies indicate that several variables like dogmatism, intolerance for ambiguity and disorder, fear of threat, and death anxiety are more closely tied to conservatives [6], [9], [30], [26], [4]. Some of these variables (e.g., fear of threat) suggest that negativity has a special value for conservatives, and recent research has indeed found ideology-based asymmetrical reactions to positive and negative stimuli, both in terms of physiological reactions [14], and intended strategies while exploring novel stimuli [15]. In the current work we explored the role of basic attentional processes, hypothesizing that people embracing conservative as compared to liberal views of the world would also display attentional mechanisms that prioritize the processing of negative information appearing in the environment. Results from three studies consistently support the existence of ideology-based differences in the automatic allocation of attentional resources. Specifically, Experiment 1 demonstrated that negative stimuli were more likely to grab the attention of conservatives, interfering with the execution of the primary task they had to perform (i.e., color-naming). Results from Experiment 2 and 3 further evidenced that ideology was related to spatial attention, and conservatives were more likely to quickly direct their attention toward negative images. Moreover, Experiment 3 clearly demonstrated that these effects remain significant even controlling for other relevant motivational factors, such as need for closure and need for cognition, thus providing further support to the hypothesis that uncertainty avoidance and threat management are two largely independent motivational factors associated with conservativism [31]. In the current studies, responses to positive and negative stimuli were contrasted and this does not allow us to clearly determine whether findings are primarily driven by conservatives' attentional bias towards negative stimuli or liberals' bias towards positive stimuli. For instance, in the Dot-Probe task employed in Experiment 2 and 3 the prioritization of one kind of stimuli necessarily implies a decreased attention to the other kind of stimuli. In the Stroop task employed in Experiment 1 responses to positive and negative stimuli were independent, and results are more in line with the idea that ideological orientation is more strongly related to responses toward negative than positive stimuli. However, although the present studies strongly support the hypothesis of ideology-based attentional asymmetries in the processing of valenced information, future research will have to compare responses to neutral and affectively-laden items and assess whether responses toward positive stimuli are also associated with ideological orientations.

Thanks to attentional processes people filter the incoming information and left-right ideological differences appear to shape these early automatic processes. As a consequence, conservatives, as compared to liberals, may indeed build up discrepant representations of the world with the former being more biased toward negativity. The outcome of this automatic selective attention for threatening information, in turn, may then further increase the motivation to embrace ideological conservatism as a way to manage uncertainty and threat [4], [32]–[34].

Ideological identification permeates our daily personal and social life. The key message is that this influence appears to emerge at very early stages of stimulus processing, indicating that negative information exerts a stronger automatic attention-grabbing power in the case of political conservatives, as compared to liberals. Thus, basic attentional processes differentiate right- and left-wingers, and they may represent one of the cognitive underpinnings creating and sustaining ideology-based different perceptions of the outside world.
